# Defining “Continuous Deep Sedation” Using Treatment Protocol: A Proposal Article

**DOI:** 10.1089/pmr.2021.0058

**Published:** 2022-02-08

**Authors:** Tatsuya Morita, Kengo Imai, Masanori Mori, Naosuke Yokomichi, Satoru Tsuneto

**Affiliations:** ^1^ Department of Palliative and Supportive Care, Seirei Mikatahara General Hospital, Hamamatsu, Shizuoka, Japan.; ^2^ Seirei Hospice, Seirei Mikatahara General Hospital, Hamamatsu, Shizuoka, Japan.; ^3^ Division of Palliative Medicine, Kyoto University, Kyoto, Japan.

**Keywords:** continuous use of sedatives, deep sedation, end-of-life care, palliative care, palliative sedation

## Abstract

***Context:*** Continuous deep sedation (CDS) is regarded as a far-reaching form of sedative use for symptom control, but there are no established uniform definitions.

***Objectives:*** To propose types of sedative use related to CDS using treatment protocols with three parameters: documented treatment goals, rapidity of dose titration, and planned duration of treatment.

***Methods:*** Opinion article.

***Results:*** We propose four types of sedative use potentially related to CDS: (1) proportional sedation (treatment goal is symptom relief with regular monitoring to maximize patient communication, not a decrease in consciousness; with gradual use of sedatives; there is a chance to cease sedatives), (2) rapid proportional sedation (treatment goal is symptom relief with a rapid loading phase, followed by regular monitoring to maximize patient communication; there is a chance to cease sedatives), (3) deep sedation with a chance of cessation (deep sedation intended initially, followed by regular assessments of appropriateness of treatment goal; there is a chance to cease sedatives), and (4) continuous deep sedation until death (deep sedation indicated from initiation and maintained until death).

***Conclusion:*** This article proposes an idea that the use of treatment protocols that visualize treatment goals, rapidity of dose titration, and planned duration of treatment may help understand the existing variations in sedative use over the world. The use of treatment protocols in the same way when defining a medical treatment in other specialty fields might clear up the current confusion about the use of sedatives.

## Introduction

Terminally ill patients experience various distressing symptoms, and palliative sedation is sometimes required for severe suffering refractory to standard palliative care measures.^1,2^ Common definitions of palliative sedation include a statement that the use of sedative medications is intended to induce a state of decreased or absent awareness to relieve the intractable suffering of dying patients.^3–12^ Continuous deep sedation (CDS) is regarded as a far-reaching form of palliative sedation, but the lack of established uniform definitions leads to confusion in the medical literature.^13^

Empirical studies suggest that CDS has marked heterogeneity. Recent conceptual and empirical studies have suggested that there are potentially two different types of palliative sedation worldwide^14–18^: (1) proportional sedation (i.e., sedatives are progressively increased according to the level of symptom palliation achieved: unconsciousness is only induced if palliation of suffering is not possible); otherwise (2) rapid deep sedation (i.e., sedatives are rapidly increased until the patient is unconscious and then they are often maintained at that level until death). If we accept this distinction, there are two types of deep sedation: one is a result of proportional sedation, and the other is an intended deep sedation from initiation.

Furthermore, the concept of continuous deep sedation until death (CDSUD: maintained deep sedation until death) was referred to in some studies,^19–24^ but it is unclear whether maintaining deep sedation is intended treatment from the initiation or a result of proportional sedation: some of them regard CDS as a result of proportional sedation for symptom relief,^19–22^ whereas some regard CDSUD as an intentional act to keep patients unconsciousness from initiation until death.^23,24^ More recently, a distinct form of CDS, deep sedation to be initiated simultaneously when life-sustaining treatment is withdrawn and maintained until death, was legalized in France.^25,26^

The intention of physicians has been regarded as an important component to approve CDS ethically and legally.^3–12^ The traditional view based on the double effect theory stresses that physicians can intend symptom relief on using sedatives, but that they should not directly intend to decrease a patient's consciousness.^27^ This is because, when the principle of double effects is applied in the practice of sedation and a decrease in consciousness is viewed as a “bad effect,” a physician should not intend to decrease consciousness, and symptom relief should not be achieved through decreased consciousness.^27^

In contrast, a recent international survey revealed a divergent attitude toward physicians' intention regarding the use of sedatives. U.K. physicians were less likely to report that their intention on the continuous use of sedatives was a decrease in consciousness and !unconsciousness, compared with Italian, German, Dutch, Belgian, and Japanese physicians: 9% versus 30%–48%, 4% versus 11%–32%, respectively.^18^ Similarly, United Kingdom and Japanese physicians were less likely to report that the common treatment goal was unconsciousness, compared with Italian, Dutch, and Belgian physicians: 22%–27% versus 54%–72%, respectively.^18^

In addition, a U.S. survey reported that 85% of U.S. physicians considered unconsciousness as an acceptable side effect of sedation but that it should not be directly intended.^28^ These findings are in line with the statements that U.K. and U.S. palliative care specialists stress the proportional use of sedatives as a measure of symptom control, and generally disagree that the intention of sedation includes a decrease in consciousness.^29–32^

Measuring a physician's intention is difficult, and we assume that a potential method to decrease the ambiguity in clinical practice may be visualizing treatment protocols with treatment goals documented using validated tools, instead of a physician's intention. To date, several studies have addressed the effectiveness of treatment protocols for sedative use in palliative care settings.^33–36^ A Japanese guideline followed by empirical studies is trying to distinguish between proportional sedation and CDS using visualized treatment protocols on the assumption that there are two types of sedative use in practice.^7,33,34^

A multicenter study revealed that the treatment protocols well reflected the intention of treatment: a proportional sedation protocol achieved satisfactory symptom relief while maintaining some patients' consciousness, and a deep sedation protocol achieved good symptom relief while the majority of patients lost consciousness.^34^ Visualizing how to use sedatives with a documented treatment goal may thus be a promising method to clarify clinical practice of sedative use. Such visualization may be also valuable in quality assurance through standardization.^37^

The aim of this conceptual article was to propose four types of sedative use related to CDS using treatment protocols. IRB approval was waived according to the national guideline on human research.

## Overview

We propose four types of sedative use potentially related to CDS using three parameters (Table 1): treatment goals described in the protocols (symptom relief vs. deep sedation), rapidity of dose titration (gradual vs. rapid with loading phase), and planned duration of treatment (during intense symptoms vs. until death). We adopted the concept of the treatment goal, instead of a physician's intention, because the treatment goal can be measured using validated tools, such as symptom intensity or levels of consciousness (e.g., Richmond Agitation-Sedation Scale [RASS]).

**Table 1.** Four Types of Sedative Use Potentially Related to Continuous Deep Sedation

**Types**	**Treatment goal described**	**Rapidity of dose titration**	**Planned duration of treatment**
Proportional sedation	Symptom relief	Gradual	During intense symptoms (there is a chance to cease sedatives)
Rapid proportional sedation	Symptom relief	Rapid	
Deep sedation with a chance of cessation	Deep sedation	Rapid	
Continuous deep sedation until death	Deep sedation	Rapid	Until death (there is no chance to cease sedatives)

The rapidity of dose titration is characterized as gradual or rapid, and the difference was visualized by the presence or absence of a loading phase. The planned duration of treatment was categorized as during intense symptoms or until death: the former means a patient has a chance to cease sedatives, but the latter means no chance to cease sedatives. In the protocols, differences are visualized by the presence or absence of regular monitoring to explore any chance of changes in the treatment goal from deep sedation to proportional sedation.

The four types of sedative use are tentatively named as (1) proportional sedation (treatment goal is symptom relief, not a decrease in consciousness, with a gradual increase in the dose, with a chance to cease sedatives), (2) rapid proportional sedation (treatment goal is symptom relief with a rapid loading phase, with a chance to cease sedatives), (3) deep sedation with a chance of cessation (deep sedation intended initially, with a chance to change the treatment goal from deep sedation to proportional sedation, potentially resulting in the cessation of sedatives), and (4) CDSUD (deep sedation indicated from initiation and maintained until death).

## Four Types of Sedative Use

### Proportional sedation

**FIG. 1.**
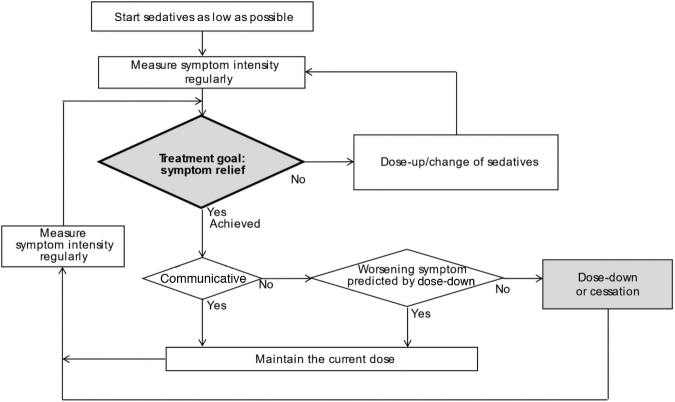
Proportional sedation. The treatment goal is symptom relief, not a decrease in consciousness; with a gradual increase in the dose there is a chance to cease sedatives.

Proportional sedation means the proportional use of sedatives with a low starting dose followed by careful dose adjustment (Fig. 1). Deep sedation is not a treatment goal described in the protocol. The treatment goal is symptom relief, and regular monitoring is vital to explore any chance to decrease the dose of sedatives, even cessation, to maximize patient communication. If patients are heavily sedated beyond symptom relief, the dose of sedatives is eventually reduced within the range of preventing worsening symptoms. If deep sedation is required, this CDS can be interpreted as a result of efforts to achieve symptom control following the proportional sedation protocol; that is, CDS is interpreted as a result of proportional sedation.

Empirically, the proportional sedation protocol led 69% goal achievement (i.e., symptom relief) four hours after initiation, with a mean of 0.8 in the Support Team Assessment Schedule (STAS) and −0.7 on the modified RASS; deep sedation was induced in 31% as a result.^34^ The fact that a relative minority of patients lost consciousness confirmed that the goal of the proportional sedation protocol is symptom relief and not deep sedation itself. This type of sedative use will be well accepted, as this may be regarded as a part of standard palliative care, like opioid titration.^30^

### Rapid proportional sedation

**FIG. 2.**
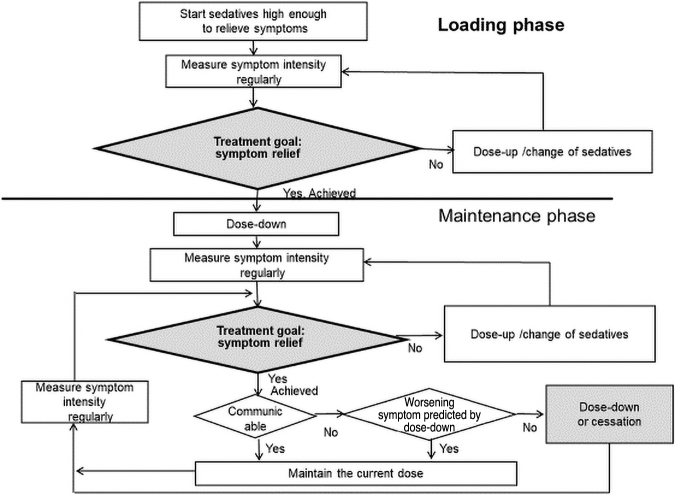
Rapid proportional sedation. The treatment goal is symptom relief with a rapid loading phase; there is a chance to cease sedatives.

Rapid proportional sedation is a form of proportional sedation, and thus the treatment goal is symptom relief, and regular monitoring is vital to explore the chance to decrease the dose of sedatives and cessation to maximize patient communication (Fig. 2). The difference from the proportional sedation protocol is the presence of a loading phase; for patients who have already been sedated due to the effects of prior medications such as respite sedation, the loading phase can be skipped.

Rapid proportional sedation is usually indicated for patients in an emergency situation, such as bleeding, suffocation, or intense dyspnea, and may be indicated for some patients at home where rapid alleviation within short time intervals is practically required. Sedatives are used at a higher dose than in typical proportional sedation, but the treatment goal is not to decrease consciousness or unconsciousness itself.

### Deep sedation with a chance of cessation

**FIG. 3.**
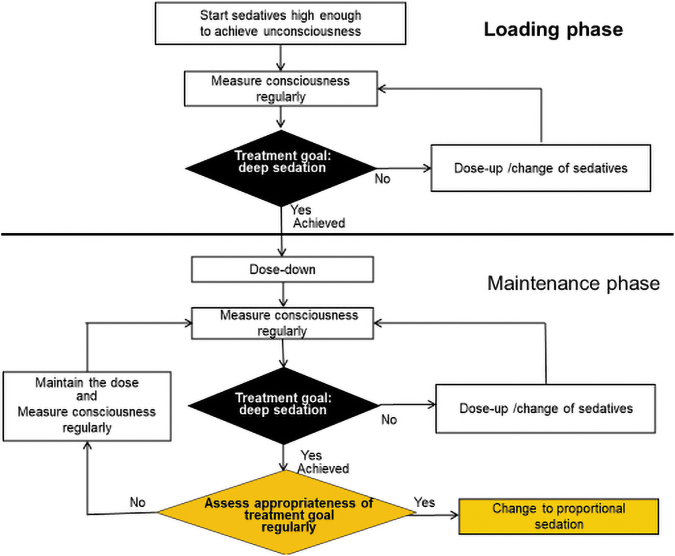
Deep sedation with a chance of cessation. Deep sedation intended initially; there is a chance of dose reduction and cessation, because the appropriateness of the treatment goal is assessed regularly and treatment protocol can be changed to proportional sedation (there is a chance to cease sedatives).

Deep sedation with a chance of cessation means deep sedation induced from initiation but with a chance of changing the treatment goal to proportional sedation (Fig. 3). The initial treatment goal is deep sedation (e.g., RASS ≤−4 or unconsciousness), and this protocol requires deep sedation during time periods when the patient's suffering does not improve. At the same time, repeated regular assessment of whether the treatment goal is appropriate is needed to explore any chance to change the treatment goal (i.e., change to proportional sedation), and then there is a chance to decrease the dose of sedatives or cessation.

An empirical study revealed that this protocol led to 83% goal achievement (i.e., deep sedation) four hours after initiation, with a mean of 0.3 in STAS and −4.2 on RASS.^34^ Of note is that, in some patients (3/7 at 24 hours), the deep sedation protocol was discontinued because patients achieved adequate symptom relief before sedation reached a deep level.^34^ The fact that not all patients reached deep sedation with this protocol suggests that physicians who state that they intend to induce deep sedation may actually intend just symptom relief, even when using the deep sedation protocol.

Therefore, this type of sedative use may be essentially the same as rapid proportional sedation. For physicians who believe that they should not directly intend to make patients unconsciousness, this type of sedation would not be acceptable; however, the fact that a considerable number of the physicians (e.g., 22%–72% in each country in an international survey) state that the treatment goal of sedative use is unconsciousness indicates that this type of sedation does exist in current clinical practice over the world.^18^

### Continuous deep sedation until death

**FIG. 4.**
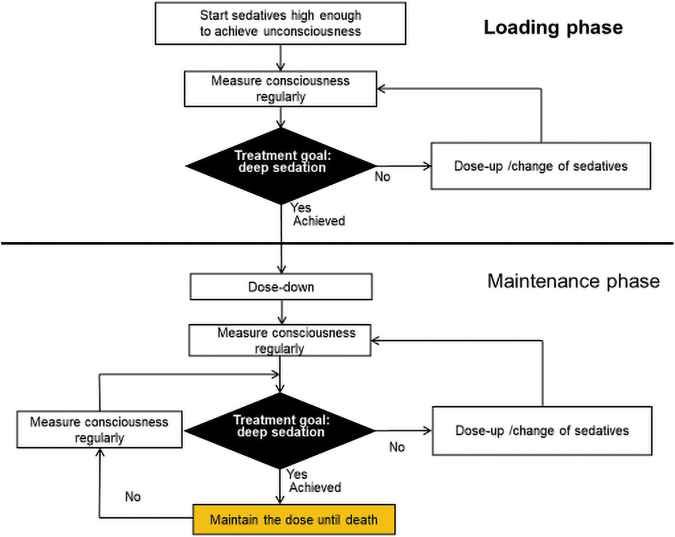
Continous deep sedation until death. Deep sedation indicated from initiation and maintained until death (there is no chance to cease sedatives).

Continuous deep sedation until death means deep sedation indicated from initiation and maintained until death as a planned medical procedure (Fig. 4). A different point from deep sedation with a chance of cessation is the lack of repeated monitoring to explore any chance to change the treatment goal, resulting in no chance of recovery. The ultimate aim is to maintain deep sedation until death, and patients have no chance to cease sedatives. Deep sedation initiated simultaneously with withdrawal of life-sustaining treatment legalized in France may be categorized into this type of sedative use.^25,26^ Some insist that no chance of changing the treatment goal and no chance of discontinuing sedatives may be inappropriate as a palliative care measure.

For example, the Japanese clinical guideline defines continuous sedation as “sedation in which a reduced level of consciousness is maintained without specifying plans to discontinue,” and prohibits deep sedation until death without regular evaluation of its necessity.^6,7^ In this guideline, eventual deep sedation until death is interpreted as a result of the fact that repeated assessments identified no appropriate timing for the withdrawal of sedation due to continuing suffering (i.e., maintaining deep sedation until death was not planned from the initiation). CDSUD as planned in the initial phase of sedation may be an essentially separate medical practice from the other three types of sedative use that involve a theoretical chance of recovery on the basis of regular assessment of the necessity of deep sedation.

## Discussion

This article proposes the use of treatment protocols to understand the current practice of sedative use using three parameters: treatment goal described in the protocols (symptom relief vs. deep sedation), rapidity of dose titration (gradual vs. rapid with loading phase), and planned duration of treatment (during intense symptoms vs. until death). Our initial proposal includes (1) proportional sedation, (2) rapid proportional sedation, (3) deep sedation with a chance of cessation, and (4) continuous deep sedation until death.

One probable critique is the argument that sedatives should be used proportionally in all settings with the intention of symptom relief (should not directly intend to decrease consciousness or unconsciousness), and thus there is only one type of sedation: proportional sedation.^29–31^ This may be appropriate and true, but empirical studies suggest that there are variations observed in actual clinical practice in treatment goals (symptom relief vs. deep sedation), the rapidity of dose titration (start sedatives as low as possible vs. high enough to achieve goal and then decrease), and planned duration of treatment (whether maintaining sedation until death is planned).^14,15,18,25,26,38^

U.K. physicians reported that their intention on the continuous use of sedatives was a decrease in consciousness and unconsciousness in 4%–9%, whereas these values among Italian, German, Dutch, Belgian, and Japanese physicians were 30%–48% and 11%–32%, respectively.^18^ Unconsciousness as a treatment goal of sedative use was reported in 22%–27% of U.K. and Japanese physicians and 54%–72% of Italian, Dutch, and Belgian physicians.^18^ The percentages of physicians who reported they started sedatives sufficiently high were 69%–72% in Belgium and Italy, 41%–54% in the Netherlands and Germany, and 22%–27% in the United Kingdom and Japan.^18^

A qualitative study suggested that sedatives were often used to maintain induced unconsciousness until death in the Netherlands and Belgium,^14,15^ and 38% of Japanese palliative care specialists surveyed in 2016 reported that they intended to maintain unconsciousness until death.^38^ Our attempt is, therefore, to classify sedative use in current practice on the basis of these parameters: treatment goals, rapidity of dose titration, and planned duration of treatment, not to discuss how sedatives should be used.

The proposal of this article is tentative, and should be regarded as a preliminary idea: there are several limitations to be considered. First, not all types of sedative use were tested in empirical studies: only two types of protocols (proportional sedation and deep sedation with a chance of cessation) were tested in a single country.^33,34^ Second, this proposal addresses only the continuous use of sedatives, and other types of sedative use such as respite sedation, especially long-term respite sedation (e.g., 48 hours), are beyond the scope of this study. Third, the combination of sedative use and withdrawal of nutrition and hydration was not considered on the classification of types of sedative use. Finally, names and classifications themselves for each type of sedative use are tentative, and further discussion is needed to reach consensus.

We propose an idea involving the use of treatment protocols that visualize treatment goals, rapidity of dose titration, and planned duration of treatment to help understand why the discussion about sedative use is confused. The use of defined treatment protocols may help understand the existing variations in sedative use over the world. Our aim is to facilitate discussion about the appropriate use of sedatives, and not to propose a definite definition of sedation. Further discussion is needed on how to define sedative use from an international perspective.

## Abbreviations Used

CDS: continuous deep sedation
CDSUD: continuous deep sedation until death
STAS: Support Team Assessment Schedule
